# 5,5′-Dimeth­oxy-2,2′-[(pentane-1,5-diyl­dioxy)bis­(nitrilo­methyl­idyne)]diphenol

**DOI:** 10.1107/S1600536809014986

**Published:** 2009-04-30

**Authors:** Yin-Xia Sun, Li Li, Wen-Kui Dong, Jian-Chao Wu, Jun-Feng Tong

**Affiliations:** a School of Chemical and Biological Engineering, Lanzhou Jiaotong University, Lanzhou 730070, People’s Republic of China

## Abstract

The mol­ecule of the title compound, C_21_H_26_N_2_O_6_, which lies across a crystallographic inversion centre, crystallizes with two unique half-molecules in the symmetric unit and adopts a linear configuration and the imino group is coplanar with the aromatic ring, making a dihedral angle of 3.26 (3)°. Strong intra­molecular O—H⋯N and weak inter­molecular O—H⋯O and C—H⋯O hydrogen bonds and weak inter­molecular π–π stacking inter­actions [centroid–centroid distance = 4.419 (2) Å]establish an infinite three-dimensional supra­molecular structure.

## Related literature

For the properties and uses of salen-type compounds, see: Lacroix (2001 ▶); Nishijo *et al.* (2006 ▶); Onda *et al.* (2007 ▶); Sun *et al.* (2004 ▶). For the structures of free salen-type compounds, see: Akine *et al.* (2005 ▶). For related structures, see: Dong *et al.* (2008*a* ▶,*b* ▶, 2009 ▶).
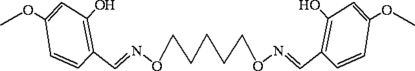


## Experimental

### 

#### Crystal data

C_21_H_26_N_2_O_6_
*M* *_r_* = 402.44
Triclinic, 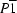

*a* = 7.3324 (15) Å
*b* = 7.6214 (17) Å
*c* = 20.372 (3) Å
α = 81.525 (1)°
β = 89.928 (2)°
γ = 67.870 (1)°
*V* = 1041.2 (3) Å^3^
*Z* = 2
Mo *K*α radiation
μ = 0.09 mm^−1^
*T* = 298 K
0.43 × 0.28 × 0.14 mm

#### Data collection

Siemens SMART 1000 CCD area-detector diffractometer
Absorption correction: multi-scan (*SADABS*; Sheldrick, 1996 ▶) *T*_min_ = 0.961, *T*_max_ = 0.987
5481 measured reflections
3618 independent reflections
1641 reflections with *I* > 2σ(*I*)
*R*_int_ = 0.028

#### Refinement

*R*[*F* ^2^ > 2σ(*F* ^2^)] = 0.052
*wR*(*F* ^2^) = 0.124
*S* = 1.01
3618 reflections
264 parameters
H-atom parameters constrained
Δρ_max_ = 0.15 e Å^−3^
Δρ_min_ = −0.18 e Å^−3^

### 

Data collection: *SMART* (Siemens, 1996 ▶); cell refinement: *SAINT* (Siemens, 1996 ▶); data reduction: *SAINT*; program(s) used to solve structure: *SHELXS97* (Sheldrick, 2008 ▶); program(s) used to refine structure: *SHELXL97* (Sheldrick, 2008 ▶); molecular graphics: *SHELXTL* (Sheldrick, 2008 ▶); software used to prepare material for publication: *SHELXTL*.

## Supplementary Material

Crystal structure: contains datablocks global, I. DOI: 10.1107/S1600536809014986/hg2502sup1.cif

Structure factors: contains datablocks I. DOI: 10.1107/S1600536809014986/hg2502Isup2.hkl

Additional supplementary materials:  crystallographic information; 3D view; checkCIF report

## Figures and Tables

**Table 1:** Hydrogen-bond geometry (Å, °)

*D*—H⋯*A*	*D*—H	H⋯*A*	*D*⋯*A*	*D*—H⋯*A*
O3—H3⋯N1	0.82	1.90	2.610 (3)	144
O5—H5⋯N2	0.82	1.90	2.628 (3)	147
O3—H3⋯O3^i^	0.82	2.68	3.045 (3)	109
C2—H2*A*⋯O3^ii^	0.97	2.58	3.533 (4)	168
C19—H19⋯O5^ii^	0.93	2.44	3.300 (4)	154

Symmetry codes: (i) 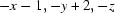; (ii) 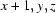.

**Fig. 1.**
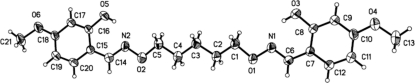
The molecular structure of the title compound with atom numbering scheme [Symmetry codes: #1 - x + 1,-y + 1,-z + 1]. Displacement ellipsoids for non-hydrogen atoms are drawn at the 30% probability level.

**Fig. 2.**
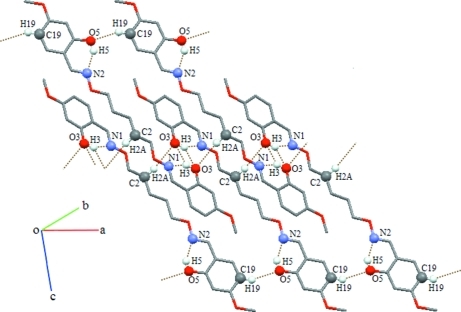
Part of the supramolecular structure of the title compound. Intra- and intermolecular hydrogen bonds are shown as dashed lines.

## supplementary crystallographic information

### Comment

Salen-type compound and its derivatives are among the most prevalent mixed-donor ligands in the field of modern coordination chemistry in which there has been growing interest, mainly because of their interesting and important properties, including optical features (Lacroix, 2001), catalytic activity in hydration of acrylonitrile (Onda *et al.*, 2007) and magnetic properties (Nishijo *et al.*, 2006). They can also be used as elemental building blocks for construction of supramolecular structures *via* intermolecular hydrogen bonding or short contact interaction (Sun *et al.*, 2004). Many salen-type complexes have been structurally characterized, but only a relatively small number of free salen-type compounds have had their X-ray structures reported (Akine *et al.*, 2005). In order to extent our work (Dong *et al.*, 2008*a*) on structural characterization of salen-type bisoxime compounds, we reported the synthesis and structure of the title compound in this paper in Fig. 1.

The molecule of the title salen-type bisoxime compound lies across a crystallographic inversion centre and adopts a linear configuration with respect to the azomethine C=N bonds. The dihedral angle formed by the two benzene rings is 23.4 (2) °, and the imino group is coplanar with the aromatic ring. This structure is different from our previous work reported in which the molecules assume W-shaped configuration (Dong *et al.*, 2008*a*) or E configuration (Dong *et al.*, 2008*b*).

There are two strong intramolecular O—H···N hydrogen bonds involving the hydroxy group and oxime N atoms in each molecule. In the crystal structure, intermolecular C—H···O and O—H···O hydrogen bonds link the each molecule to three others, and weak intermolecular π-π stacking interaction between the neighbouring benzene rings (the inter-molecular plane-to-plane dihedral angle along *b* axis is 0.48 (4) °). Thus, an infinite three-dimensional supramolecular structure is established (Fig. 2).

### Experimental

5,5'-Dimethoxy-2,2'-[(pentane-1,5-diyldioxy)bis(nitrilomethylidyne)]diphenol was synthesized according to an analogous method reported earlier (Dong *et al.*, 2009). To an ethanol solution (10 ml) of 4-methoxy-2-hydroxybenzaldehyde (304.3 mg, 2.00 mmol) was added an ethanol solution (6 ml) of 1,5-bis(aminooxy)pentane (134.2 mg, 1.00 mmol). The reaction mixture was stirred at 328 K for 5 h. The formed precipitate was separated by filtration, and washed successively with ethanol and ethanol-hexane (1:4), respectively. The product was dried under vacuum to yield 204.2 mg of the title compound. Yield, 51.8%. mp. 349–350 K. Anal. Calc. for C_21_H_26_N_2_O_6_: C, 62.67; H, 6.51; N, 9.96. Found: C, 62.79; H, 6.68; N, 6.83.

Colorless block-like single crystals suitable for X-ray diffraction studies were obtained after about two months by slow evaporation from an ethanol solution of the title compound.

### Refinement

Non-H atoms were refined anisotropically. H atoms were treated as riding atoms with distances C—H = 0.97 (CH_2_), 0.93 Å (CH), O—H = 0.82 Å and *U*_iso_(H) = 1.2 *U*_eq_(C) and 1.5 *U*_eq_(O).

### Crystal data

**Table:**

C_21_H_26_N_2_O_6_	*Z* = 2
*M**_r_* = 402.44	*F*(000) = 428
Triclinic, *P*1	*D*_x_ = 1.284 Mg m^−^^3^
Hall symbol: -P 1	Mo *K*α radiation, λ = 0.71073 Å
*a* = 7.3324 (15) Å	Cell parameters from 974 reflections
*b* = 7.6214 (17) Å	θ = 2.9–23.1°
*c* = 20.372 (3) Å	µ = 0.09 mm^−^^1^
α = 81.525 (1)°	*T* = 298 K
β = 89.928 (2)°	Block-like, colorless
γ = 67.870 (1)°	0.43 × 0.28 × 0.14 mm
*V* = 1041.2 (3) Å^3^	

### Data collection

**Table:**

Siemens SMART 1000 CCD area-detector diffractometer	3618 independent reflections
Radiation source: fine-focus sealed tube	1641 reflections with *I* > 2σ(*I*)
graphite	*R*_int_ = 0.028
φ and ω scans	θ_max_ = 25.0°, θ_min_ = 2.0°
Absorption correction: multi-scan (*SADABS*; Sheldrick, 1996)	*h* = −8→8
*T*_min_ = 0.961, *T*_max_ = 0.987	*k* = −9→8
5481 measured reflections	*l* = −24→15

### Refinement

**Table:**

Refinement on *F*^2^	Primary atom site location: structure-invariant direct methods
Least-squares matrix: full	Secondary atom site location: difference Fourier map
*R*[*F*^2^ > 2σ(*F*^2^)] = 0.052	Hydrogen site location: inferred from neighbouring sites
*wR*(*F*^2^) = 0.124	H-atom parameters constrained
*S* = 1.01	*w* = 1/[σ^2^(*F*_o_^2^) + (0.0371*P*)^2^] where *P* = (*F*_o_^2^ + 2*F*_c_^2^)/3
3618 reflections	(Δ/σ)_max_ < 0.001
264 parameters	Δρ_max_ = 0.14 e Å^−^^3^
0 restraints	Δρ_min_ = −0.18 e Å^−^^3^

### Special details

**Table:**

Geometry. All e.s.d.'s (except the e.s.d. in the dihedral angle between two l.s. planes) are estimated using the full covariance matrix. The cell e.s.d.'s are taken into account individually in the estimation of e.s.d.'s in distances, angles and torsion angles; correlations between e.s.d.'s in cell parameters are only used when they are defined by crystal symmetry. An approximate (isotropic) treatment of cell e.s.d.'s is used for estimating e.s.d.'s involving l.s. planes.
Refinement. Refinement of *F*^2^ against ALL reflections. The weighted *R*-factor *wR* and goodness of fit *S* are based on *F*^2^, conventional *R*-factors *R* are based on *F*, with *F* set to zero for negative *F*^2^. The threshold expression of *F*^2^ > σ(*F*^2^) is used only for calculating *R*-factors(gt) *etc*. and is not relevant to the choice of reflections for refinement. *R*-factors based on *F*^2^ are statistically about twice as large as those based on *F*, and *R*- factors based on ALL data will be even larger.

### Fractional atomic coordinates and isotropic or equivalent isotropic displacement parameters (Å^2^)

**Table:**

	*x*	*y*	*z*	*U*_iso_*/*U*_eq_	
N1	−0.1725 (3)	0.8216 (3)	−0.07342 (10)	0.0615 (6)	
N2	0.8214 (3)	0.4398 (3)	0.28534 (10)	0.0657 (6)	
O1	0.0265 (3)	0.7497 (3)	−0.05195 (8)	0.0749 (6)	
O2	0.7701 (3)	0.4917 (3)	0.21730 (8)	0.0800 (6)	
O3	−0.5543 (2)	0.9488 (3)	−0.06470 (8)	0.0882 (7)	
H3	−0.4405	0.9249	−0.0516	0.132*	
O4	−0.9588 (3)	1.0916 (3)	−0.25645 (9)	0.0917 (7)	
O5	0.8015 (2)	0.3232 (3)	0.41215 (8)	0.0891 (7)	
H5	0.7610	0.3633	0.3732	0.134*	
O6	1.3399 (3)	0.2041 (3)	0.55807 (9)	0.0906 (7)	
C1	0.0438 (4)	0.7272 (4)	0.01908 (12)	0.0678 (8)	
H1A	−0.0151	0.6387	0.0387	0.081*	
H1B	−0.0235	0.8497	0.0339	0.081*	
C2	0.2584 (4)	0.6508 (4)	0.03966 (11)	0.0699 (8)	
H2A	0.3156	0.7376	0.0173	0.084*	
H2B	0.3220	0.5279	0.0248	0.084*	
C3	0.3029 (4)	0.6250 (4)	0.11341 (11)	0.0651 (8)	
H3A	0.2570	0.5291	0.1357	0.078*	
H3B	0.2305	0.7449	0.1292	0.078*	
C4	0.5200 (4)	0.5643 (4)	0.13208 (12)	0.0662 (8)	
H4A	0.5930	0.4465	0.1151	0.079*	
H4B	0.5651	0.6621	0.1109	0.079*	
C5	0.5645 (4)	0.5332 (4)	0.20517 (12)	0.0692 (8)	
H5A	0.4861	0.6473	0.2233	0.083*	
H5B	0.5322	0.4272	0.2266	0.083*	
C6	−0.1984 (4)	0.8436 (4)	−0.13669 (12)	0.0607 (7)	
H6	−0.0899	0.8147	−0.1628	0.073*	
C7	−0.3954 (4)	0.9133 (4)	−0.16794 (12)	0.0528 (7)	
C8	−0.5646 (4)	0.9604 (4)	−0.13165 (12)	0.0599 (8)	
C9	−0.7468 (4)	1.0168 (4)	−0.16277 (13)	0.0729 (9)	
H9	−0.8574	1.0445	−0.1377	0.087*	
C10	−0.7693 (4)	1.0334 (4)	−0.23081 (14)	0.0633 (8)	
C11	−0.6068 (4)	0.9916 (4)	−0.26870 (13)	0.0691 (8)	
H11	−0.6201	1.0020	−0.3147	0.083*	
C12	−0.4233 (4)	0.9337 (4)	−0.23622 (12)	0.0661 (8)	
H12	−0.3132	0.9071	−0.2615	0.079*	
C13	−0.9911 (4)	1.1077 (4)	−0.32617 (14)	0.0919 (11)	
H13A	−0.9339	1.1923	−0.3489	0.138*	
H13B	−1.1303	1.1582	−0.3376	0.138*	
H13C	−0.9310	0.9834	−0.3392	0.138*	
C14	0.9944 (4)	0.4320 (4)	0.29886 (13)	0.0642 (8)	
H14	1.0649	0.4660	0.2647	0.077*	
C15	1.0837 (4)	0.3722 (4)	0.36552 (12)	0.0534 (7)	
C16	0.9873 (4)	0.3203 (4)	0.42012 (13)	0.0611 (8)	
C17	1.0779 (4)	0.2636 (4)	0.48276 (13)	0.0727 (9)	
H17	1.0124	0.2280	0.5182	0.087*	
C18	1.2649 (4)	0.2590 (4)	0.49373 (13)	0.0627 (8)	
C19	1.3648 (4)	0.3074 (4)	0.44140 (13)	0.0669 (8)	
H19	1.4922	0.3023	0.4483	0.080*	
C20	1.2727 (4)	0.3637 (4)	0.37860 (13)	0.0670 (8)	
H20	1.3402	0.3975	0.3434	0.080*	
C21	1.5276 (4)	0.2114 (4)	0.57277 (13)	0.0959 (11)	
H21A	1.6282	0.1171	0.5524	0.144*	
H21B	1.5552	0.1853	0.6200	0.144*	
H21C	1.5251	0.3366	0.5558	0.144*	

### Atomic displacement parameters (Å^2^)

**Table:**

	*U*^11^	*U*^22^	*U*^33^	*U*^12^	*U*^13^	*U*^23^
N1	0.0551 (15)	0.0683 (17)	0.0543 (15)	−0.0171 (13)	−0.0062 (11)	−0.0058 (12)
N2	0.0634 (16)	0.0769 (18)	0.0557 (15)	−0.0256 (14)	−0.0110 (12)	−0.0096 (12)
O1	0.0562 (12)	0.1062 (17)	0.0539 (12)	−0.0218 (11)	−0.0072 (9)	−0.0116 (10)
O2	0.0725 (14)	0.1078 (18)	0.0599 (12)	−0.0379 (12)	−0.0111 (10)	−0.0041 (11)
O3	0.0699 (13)	0.140 (2)	0.0495 (12)	−0.0358 (13)	0.0041 (10)	−0.0105 (11)
O4	0.0660 (14)	0.128 (2)	0.0742 (14)	−0.0307 (13)	−0.0134 (11)	−0.0099 (12)
O5	0.0537 (12)	0.150 (2)	0.0738 (13)	−0.0519 (13)	0.0011 (9)	−0.0124 (12)
O6	0.0790 (15)	0.132 (2)	0.0708 (14)	−0.0603 (14)	−0.0180 (11)	0.0084 (12)
C1	0.0641 (19)	0.078 (2)	0.0539 (18)	−0.0198 (17)	−0.0075 (14)	−0.0074 (15)
C2	0.0615 (19)	0.083 (2)	0.0596 (18)	−0.0226 (17)	−0.0068 (14)	−0.0071 (15)
C3	0.0636 (19)	0.066 (2)	0.0589 (18)	−0.0185 (16)	−0.0073 (14)	−0.0055 (14)
C4	0.068 (2)	0.065 (2)	0.0620 (18)	−0.0220 (16)	−0.0093 (14)	−0.0086 (14)
C5	0.064 (2)	0.072 (2)	0.0655 (19)	−0.0217 (17)	−0.0124 (15)	−0.0053 (15)
C6	0.0586 (18)	0.069 (2)	0.0511 (17)	−0.0209 (16)	0.0053 (14)	−0.0088 (14)
C7	0.0553 (18)	0.0558 (19)	0.0467 (16)	−0.0212 (15)	−0.0018 (14)	−0.0061 (13)
C8	0.0620 (19)	0.072 (2)	0.0433 (17)	−0.0251 (16)	0.0005 (14)	−0.0045 (14)
C9	0.0581 (19)	0.104 (3)	0.0542 (19)	−0.0273 (18)	0.0038 (14)	−0.0135 (16)
C10	0.0546 (19)	0.068 (2)	0.063 (2)	−0.0212 (16)	−0.0073 (16)	−0.0059 (15)
C11	0.073 (2)	0.082 (2)	0.0518 (18)	−0.0293 (18)	−0.0038 (16)	−0.0088 (15)
C12	0.0633 (19)	0.083 (2)	0.0512 (18)	−0.0259 (17)	0.0038 (14)	−0.0120 (15)
C13	0.089 (2)	0.100 (3)	0.080 (2)	−0.029 (2)	−0.0296 (18)	−0.0106 (19)
C14	0.0625 (19)	0.070 (2)	0.0630 (19)	−0.0279 (17)	0.0019 (15)	−0.0109 (15)
C15	0.0495 (17)	0.0574 (19)	0.0555 (17)	−0.0213 (14)	0.0037 (13)	−0.0139 (13)
C16	0.0417 (16)	0.079 (2)	0.0681 (19)	−0.0269 (15)	0.0022 (14)	−0.0186 (15)
C17	0.0602 (19)	0.108 (3)	0.0597 (19)	−0.0455 (18)	0.0014 (15)	−0.0068 (17)
C18	0.0563 (18)	0.073 (2)	0.0621 (19)	−0.0302 (16)	−0.0057 (15)	−0.0061 (15)
C19	0.0470 (17)	0.083 (2)	0.076 (2)	−0.0294 (16)	0.0010 (15)	−0.0134 (16)
C20	0.0539 (18)	0.085 (2)	0.069 (2)	−0.0357 (17)	0.0067 (14)	−0.0086 (16)
C21	0.075 (2)	0.123 (3)	0.097 (2)	−0.054 (2)	−0.0304 (18)	0.003 (2)

### Geometric parameters (Å, °)

**Table:**

N1—C6	1.280 (2)	C6—C7	1.449 (3)
N1—O1	1.396 (2)	C6—H6	0.9300
N2—C14	1.276 (3)	C7—C12	1.383 (3)
N2—O2	1.395 (2)	C7—C8	1.398 (3)
O1—C1	1.432 (2)	C8—C9	1.364 (3)
O2—C5	1.432 (3)	C9—C10	1.378 (3)
O3—C8	1.354 (2)	C9—H9	0.9300
O3—H3	0.8200	C10—C11	1.379 (3)
O4—C10	1.367 (3)	C11—C12	1.382 (3)
O4—C13	1.420 (3)	C11—H11	0.9300
O5—C16	1.363 (2)	C12—H12	0.9300
O5—H5	0.8200	C13—H13A	0.9600
O6—C18	1.365 (3)	C13—H13B	0.9600
O6—C21	1.431 (3)	C13—H13C	0.9600
C1—C2	1.493 (3)	C14—C15	1.441 (3)
C1—H1A	0.9700	C14—H14	0.9300
C1—H1B	0.9700	C15—C20	1.387 (3)
C2—C3	1.506 (3)	C15—C16	1.405 (3)
C2—H2A	0.9700	C16—C17	1.371 (3)
C2—H2B	0.9700	C17—C18	1.376 (3)
C3—C4	1.513 (3)	C17—H17	0.9300
C3—H3A	0.9700	C18—C19	1.377 (3)
C3—H3B	0.9700	C19—C20	1.378 (3)
C4—C5	1.490 (3)	C19—H19	0.9300
C4—H4A	0.9700	C20—H20	0.9300
C4—H4B	0.9700	C21—H21A	0.9600
C5—H5A	0.9700	C21—H21B	0.9600
C5—H5B	0.9700	C21—H21C	0.9600
			
C6—N1—O1	112.7 (2)	C9—C8—C7	120.7 (2)
C14—N2—O2	111.7 (2)	C8—C9—C10	120.9 (3)
N1—O1—C1	109.48 (18)	C8—C9—H9	119.5
N2—O2—C5	110.15 (19)	C10—C9—H9	119.5
C8—O3—H3	109.5	O4—C10—C9	115.7 (3)
C10—O4—C13	118.4 (2)	O4—C10—C11	124.0 (3)
C16—O5—H5	109.5	C9—C10—C11	120.3 (2)
C18—O6—C21	117.9 (2)	C10—C11—C12	117.9 (2)
O1—C1—C2	107.6 (2)	C10—C11—H11	121.1
O1—C1—H1A	110.2	C12—C11—H11	121.1
C2—C1—H1A	110.2	C11—C12—C7	123.3 (3)
O1—C1—H1B	110.2	C11—C12—H12	118.4
C2—C1—H1B	110.2	C7—C12—H12	118.4
H1A—C1—H1B	108.5	O4—C13—H13A	109.5
C1—C2—C3	114.5 (2)	O4—C13—H13B	109.5
C1—C2—H2A	108.6	H13A—C13—H13B	109.5
C3—C2—H2A	108.6	O4—C13—H13C	109.5
C1—C2—H2B	108.6	H13A—C13—H13C	109.5
C3—C2—H2B	108.6	H13B—C13—H13C	109.5
H2A—C2—H2B	107.6	N2—C14—C15	121.9 (2)
C2—C3—C4	113.2 (2)	N2—C14—H14	119.1
C2—C3—H3A	108.9	C15—C14—H14	119.1
C4—C3—H3A	108.9	C20—C15—C16	116.6 (2)
C2—C3—H3B	108.9	C20—C15—C14	120.6 (2)
C4—C3—H3B	108.9	C16—C15—C14	122.8 (2)
H3A—C3—H3B	107.8	O5—C16—C17	118.2 (2)
C5—C4—C3	113.1 (2)	O5—C16—C15	120.9 (2)
C5—C4—H4A	109.0	C17—C16—C15	121.0 (2)
C3—C4—H4A	109.0	C16—C17—C18	120.6 (2)
C5—C4—H4B	109.0	C16—C17—H17	119.7
C3—C4—H4B	109.0	C18—C17—H17	119.7
H4A—C4—H4B	107.8	O6—C18—C17	115.9 (2)
O2—C5—C4	108.8 (2)	O6—C18—C19	124.0 (2)
O2—C5—H5A	109.9	C17—C18—C19	120.1 (2)
C4—C5—H5A	109.9	C18—C19—C20	118.8 (2)
O2—C5—H5B	109.9	C18—C19—H19	120.6
C4—C5—H5B	109.9	C20—C19—H19	120.6
H5A—C5—H5B	108.3	C19—C20—C15	122.9 (2)
N1—C6—C7	120.5 (2)	C19—C20—H20	118.6
N1—C6—H6	119.8	C15—C20—H20	118.6
C7—C6—H6	119.8	O6—C21—H21A	109.5
C12—C7—C8	116.8 (2)	O6—C21—H21B	109.5
C12—C7—C6	120.5 (2)	H21A—C21—H21B	109.5
C8—C7—C6	122.6 (2)	O6—C21—H21C	109.5
O3—C8—C9	117.5 (2)	H21A—C21—H21C	109.5
O3—C8—C7	121.7 (2)	H21B—C21—H21C	109.5
			
C6—N1—O1—C1	−179.4 (2)	C9—C10—C11—C12	−0.1 (4)
C14—N2—O2—C5	−169.4 (2)	C10—C11—C12—C7	1.0 (4)
N1—O1—C1—C2	−179.9 (2)	C8—C7—C12—C11	−2.0 (4)
O1—C1—C2—C3	177.9 (2)	C6—C7—C12—C11	177.2 (3)
C1—C2—C3—C4	−174.9 (2)	O2—N2—C14—C15	−176.9 (2)
C2—C3—C4—C5	−178.3 (2)	N2—C14—C15—C20	179.5 (3)
N2—O2—C5—C4	−173.06 (19)	N2—C14—C15—C16	−0.6 (4)
C3—C4—C5—O2	−175.4 (2)	C20—C15—C16—O5	−179.7 (2)
O1—N1—C6—C7	178.8 (2)	C14—C15—C16—O5	0.4 (4)
N1—C6—C7—C12	−178.8 (2)	C20—C15—C16—C17	−0.3 (4)
N1—C6—C7—C8	0.4 (4)	C14—C15—C16—C17	179.8 (2)
C12—C7—C8—O3	−178.9 (2)	O5—C16—C17—C18	−179.6 (3)
C6—C7—C8—O3	1.9 (4)	C15—C16—C17—C18	0.9 (4)
C12—C7—C8—C9	2.3 (4)	C21—O6—C18—C17	−175.2 (3)
C6—C7—C8—C9	−176.9 (2)	C21—O6—C18—C19	5.0 (4)
O3—C8—C9—C10	179.5 (2)	C16—C17—C18—O6	178.8 (2)
C7—C8—C9—C10	−1.6 (4)	C16—C17—C18—C19	−1.4 (4)
C13—O4—C10—C9	−178.8 (2)	O6—C18—C19—C20	−179.0 (2)
C13—O4—C10—C11	0.9 (4)	C17—C18—C19—C20	1.2 (4)
C8—C9—C10—O4	−179.8 (3)	C18—C19—C20—C15	−0.6 (4)
C8—C9—C10—C11	0.5 (4)	C16—C15—C20—C19	0.1 (4)
O4—C10—C11—C12	−179.8 (2)	C14—C15—C20—C19	−180.0 (2)

### Hydrogen-bond geometry (Å, °)

**Table:**

*D*—H···*A*	*D*—H	H···*A*	*D*···*A*	*D*—H···*A*
O3—H3···N1	0.82	1.90	2.610 (3)	144
O5—H5···N2	0.82	1.90	2.628 (3)	147
O3—H3···O3^i^	0.82	2.68	3.045 (3)	109
C2—H2A···O3^ii^	0.97	2.58	3.533 (4)	168
C19—H19···O5^ii^	0.93	2.44	3.300 (4)	154

Symmetry codes: (i) −*x*−1, −*y*+2, −*z*; (ii) *x*+1, *y*, *z*.
